# Luteolin as a urease inhibitor: A novel therapeutic strategy against *Helicobacter pylori*

**DOI:** 10.1080/21505594.2026.2712031

**Published:** 2026-08-03

**Authors:** Xingye Wang, Dongyue Zhou, Mingran Li, Xiangri Kong, Shuang Jiang, Qingjie Li, Lin Wei, Lihong Jiang, Wu Song

**Affiliations:** a College of Traditional Chinese Medicine, Changchun University of Chinese Medicine, Changchun, China; b Jiangxi Province Key Laboratory of Traditional Chinese Medicine Pharmacology, Institute of Traditional Chinese Medicine Health Industry, China Academy of Chinese Medical Sciences, Nanchang, China; c Jiangxi Health Industry Institute of Traditional Chinese Medicine, Nanchang, China; d College of Integrated Chinese and Western Medicine, Changchun University of Chinese Medicine, Changchun, China; e Endocrinology Department, The Affiliated Hospital to Changchun University of Chinese Medicine, Changchun, China; f School of Basic Medicine, Changchun University of Chinese Medicine, Changchun, China; g Research Center of Traditional Chinese Medicine, The Affiliated Hospital to Changchun University of Chinese Medicine, Changchun, China

**Keywords:** *Helicobacter pylori*, urease, luteolin, ammonia restriction, metabolic disruption

## Abstract

The rising prevalence of antibiotic-resistant *Helicobacter pylori* (*H. pylori*) underscores the urgent need for alternative treatment strategies. By producing ammonia to neutralize gastric acid, the key virulence factor urease is essential for *H. pylori* acid tolerance, thereby representing a promising therapeutic target. In this study, we identified luteolin as a potent urease inhibitor (IC_50_ = 30.50 μg/mL) from a screening of over 200 natural compounds. Further investigation through molecular docking, dynamics simulations, cellular thermal shift assay, and enzyme kinetics studies confirmed its competitive binding to the Ni^2+^-centered catalytic site of *H. pylori* urease (HPU). Luteolin exhibited potent anti-*H. pylori* activity under standard and simulated gastric conditions, and showed low propensity for resistance development over 14 serial passages. Proteomic and metabolomic analyses revealed that luteolin inhibited HPU activity, and the consequent ammonia restriction triggered severe metabolic dysfunction, characterized by disruptions in nucleotide, amino acid biosynthesis and TCA cycle. In GES-1 cells, luteolin protected against *H. pylori*-induced damage. In a mouse model of *H. pylori*-induced peptic ulcer, luteolin treatment significantly reduced the bacterial load, with concomitant alleviation of gastric mucosal pathology and suppression of inflammatory responses. In contrast to antibiotic-induced gastric microbial dysbiosis, microbial diversity analysis indicated that luteolin treatment had minimal impact on the resident gastric microbiota. In summary, as a urease inhibitor, luteolin suppresses *H. pylori* by blocking ammonia production, thereby disrupting acid neutralization and inducing metabolic dysfunction, which collectively alleviates gastric damage and inflammation while minimizing microbiota disruption and resistance risk.

## GRAPHICAL ABSTRACT

**Figure:**
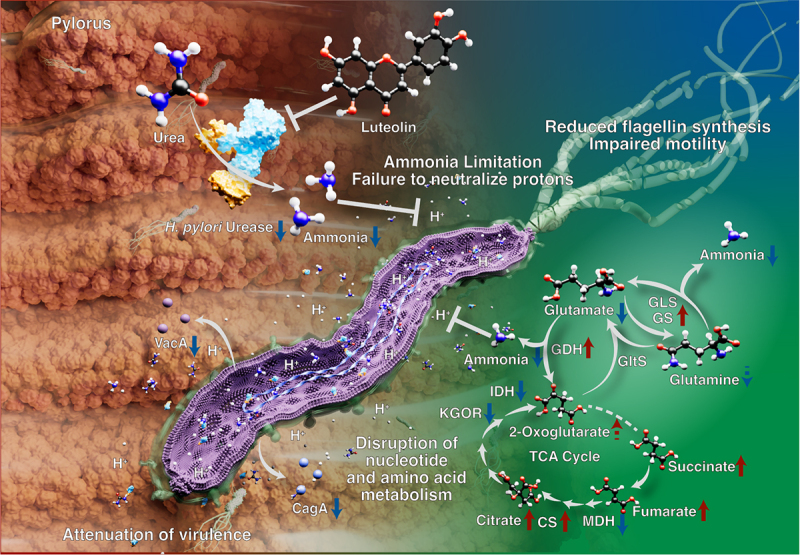

## Introduction

*Helicobacter pylori* (*H. pylori*) is a highly prevalent global pathogen, affecting approximately half of the global population [1,2]. In recent years, epidemiological evidence demonstrates a concerning rise in antibiotic resistance rates in *H. pylori*, directly compromising therapeutic outcomes [3–5]. Consequently, advancing novel non-antibiotic strategies has become an imperative to address this critical need. *H. pylori* infection, the major cause of peptic ulcers, relies on urease and flagella for gastric colonization [6]. Following colonization facilitated by urease and motility, virulence factors like CagA and VacA trigger inflammation, disrupt the mucosal barrier, and promote cellular damage linked to carcinogenesis [7]. These virulence factors are therefore pivotal determinants of *H. pylori* pathogenicity [8], making them attractive targets for novel therapeutic strategies.

*H. pylori* urease (HPU, EC 3.5.1.5) is essential to the survival and pathogenicity of *H. pylori* [9,10]. HPU catalyzes urea hydrolysis to ammonia, which neutralizes gastric acid, thereby maintaining pH homeostasis for survival [11]. The ammonia establishes an alkaline microenvironment that is both damaging to gastric epithelial cells and conducive to bacterial colonization. Simultaneously, as the primary nitrogen source for *H. pylori*, urea-derived ammonia is utilized for the biosynthesis of amino acids, purines and pyrimidines [12]. Notably, a mutant strain of *H. pylori* deficient in urease activity showed a markedly diminished capacity to colonize the murine stomach [13]. Although the FDA-approved urease inhibitor Lithostat® (acetohydroxamic acid, AHA) is effective against HPU, its severe side effects, including teratogenicity, psychoneurologic and musculo-integumentary symptoms, limit its clinical utility [14,15]. Therefore, replacing or supplementing current *H. pylori* eradication regimens with potent urease inhibitors represents a promising strategy to enhance therapeutic efficacy and overcome resistance.

Luteolin is a widely distributed natural flavonoid that possesses diverse biological activities, demonstrating considerable potential in anti-inflammatory, anticancer, antioxidant, and antibacterial applications [16–19]. Clinical studies on NeuroProtek®, a dietary flavonoid formulation containing luteolin, have established its safety profile. In a case series involving 37 children aged 4–14 y who were administered a luteolin-containing formulation for at least 4 months, no adverse effects were reported [20]. A recognized limitation of luteolin is its low oral bioavailability (~26% in rats), reflecting poor solubility, limited permeability, and extensive pre-systemic metabolism [21,22]. These features are further supported by in silico ADMET prediction (predicted oral bioavailability < 20%; ADMET lab 3.0 [23]). However, the target here, *H. pylori* urease, resides at the gastric luminal and mucosal surface rather than in the systemic circulation, and orally administered luteolin is retained in the stomach predominantly as the bioactive aglycone [24]. Limited systemic exposure therefore does not preclude local action at the site of infection.

Driven by the need for novel anti-*H. pylori* strategies beyond conventional antibiotics, we identified the natural flavonoid luteolin as a potent urease inhibitor. This study established luteolin as a promising therapeutic candidate by demonstrating its high efficacy in eradicating *H. pylori*, alleviating disease pathology, with favorable properties of minimizing disruption to the resident microbiota and reducing the propensity for resistance development. Acting as a urease inhibitor, luteolin suppresses *H. pylori* through blockade of ammonia production, thereby disrupting acid neutralization and triggering biosynthetic dysfunction.

## Materials and methods

### Bacterial strain, cell line, reagents, and culture conditions

The *H. pylori* strain ATCC 43504 (*cagA*+, *vacA*+), obtained from the American Type Culture Collection (Manassas, VA, USA), was utilized in this study. The bacteria were routinely cultured on a modified agar base (HB8646, HopeBio, China) supplemented with 7% sterile defibrinated sheep blood (1001339-1, HopeBio, China) or 10% FBS (G8003, ServiceBio, China) to meet various experimental requirements. A selective supplement (HB8646a, HopeBio, China) containing nalidixic acid, trimethoprim, vancomycin, and amphotericin B was added for the isolation and colony counting of *H. pylori* from samples. All cultures were incubated under microaerophilic environment (37°C, 90% relative humidity, 10% CO_2_, 5% O_2_, and 85% N_2_) in a triple-gas incubator (BOLV, China) for bacterial growth over 48–96 h. For liquid culture, *H. pylori* was inoculated in a modified broth (HB8647, HopeBio, China) supplemented with 10% FBS and incubated with shaking at 180 rpm on a mini shaker (NY-H1S, Enyi Technology, China) placed inside the triple-gas incubator.

The human gastric epithelial cell line (GES-1, RRID:CVCL_EQ22, STCC10401, ServiceBio, China) was cultured in DMEM-high glucose medium (G4515, ServiceBio, China), supplemented with 10% FBS (F0193, Sigma, USA) within a humidified incubator (Thermo Fisher, USA) maintained at 37 °C with 5% CO_2_.

Luteolin (purity > 98%) was purchased from Chengdu Herbpurify Co., Ltd. (Lot No. RFS-M00702004028, China). Unless otherwise specified, luteolin was dissolved in DMSO to a concentration of 20 mg/mL. To avoid potential solvent interference from DMSO in the Berthelot assay for urease activity, a 5 mg/mL stock solution was prepared using anhydrous ethanol instead, with the addition of 1 M NaOH to facilitate dissolution. This method has been verified to have almost no impact on detection efficiency.

### In silicon molecular docking and molecular dynamics study

Molecular docking was performed to predict the binding modes of compounds to HPU complex (PDB code: 1e9y) using SwissDock web service with AutoDock Vina [25]. The protein structure was prepared by adding hydrogen atoms, and removing water molecules and the co-crystallized substrate (AHA). The active site was defined as a 30 Å cubic box centered on Ni^2+^. Default parameters were used for docking, and the resulting poses were ranked based on their docking scores.

Molecular dynamics simulations were carried out using Desmond 2022.1 (Schrödinger LLC). Each system was solvated with TIP3P water and neutralized with 0.15 M NaCl. After energy minimization and equilibration, a 100 ns production simulation was performed under an NPT ensemble (300 K, 1 bar). Trajectories were saved every 100 ps for subsequent analysis. The simulation data were analyzed using the Simulation Interaction Diagram tool within Desmond.

### Biochemical analysis of urease inhibition

**Figure 1.**
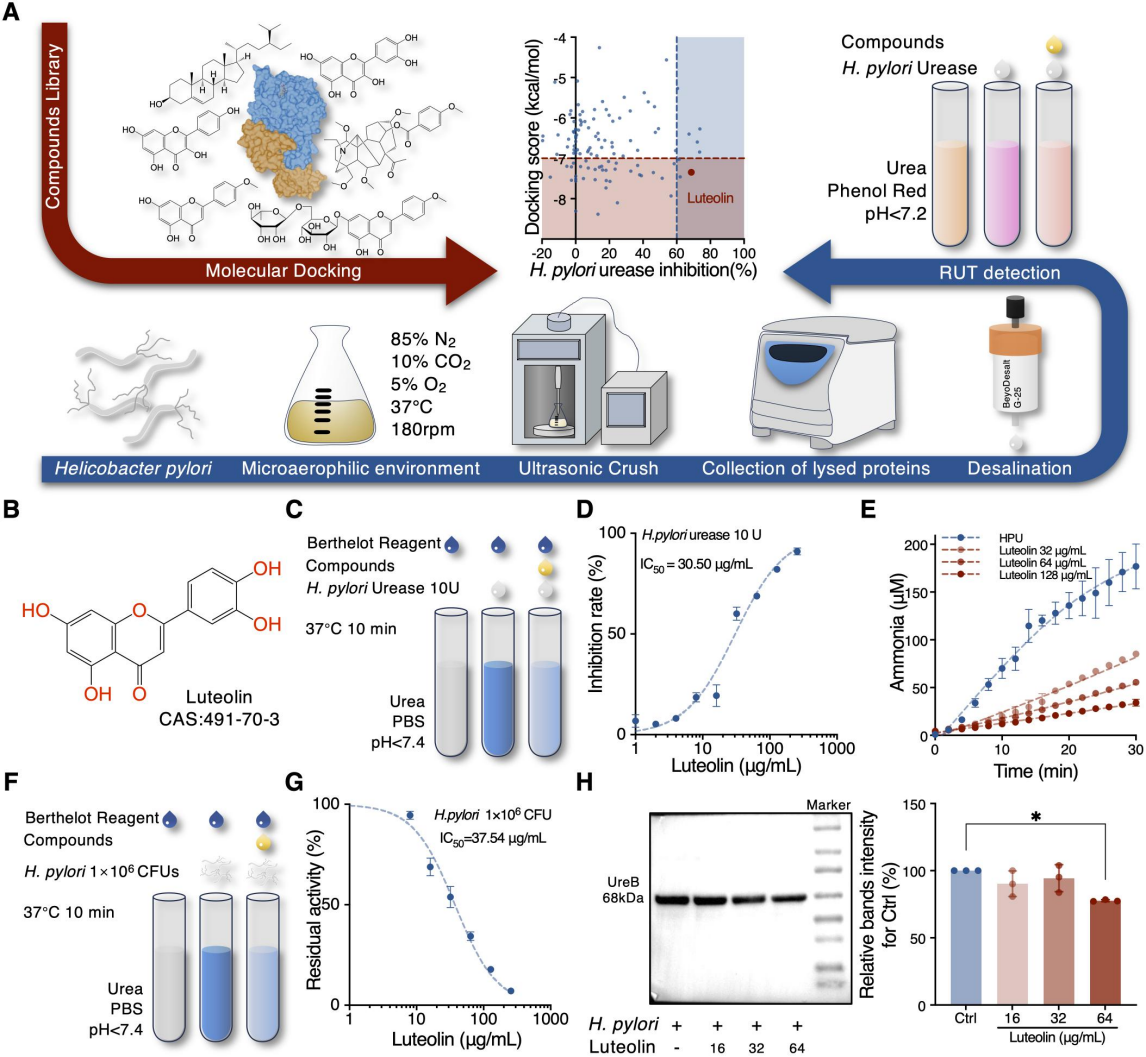
Luteolin is a potential HPU inhibitor. A. Schematic diagram of the two-stage screening strategy for HPU inhibitors, virtual screening (DS <  −7 kcal/mol) followed by experimental validation using the RUT (inhibition rate ≥ 60%). B. Chemical structure of luteolin. C-D. Anti-HPU activity of luteolin *in vitro*, with an IC_50_ value of 30.50 μg/mL. E. A standard curve for the Berthelot reaction was generated using NH_4_Cl at gradient concentrations to quantify ammonia production. F-G. Anti-HPU activity of luteolin in intact bacteria, with an IC_50_ of 37.54 μg/mL. H. Western blotting analysis of UreB expression in *H. pylori* after 24 h treatment with luteolin (*p < 0.05). Data are from three independent experiments and presented as mean ± SD.

Native HPU, used as the enzyme source for the *in vitro* inhibition assays, was prepared as outlined in Figure 1(A), following a previously described method with modifications [26]. Briefly, *H. pylori* colonies were collected with an inoculation loop, resuspended in PBS, and adjusted to a McFarland standard of 1.0. The suspension was then centrifuged at 4,000×g for 10 min at 4°C and washed twice with PBS. The pellet was stored at −80°C for at least 8 h. After thawing, the pellet was lysed by adding 3 mL of distilled water containing 1% (v/v) PMSF, followed by ultrasonication (5 s on/off pulses) for 8 min on ice. The lysate was centrifuged at 15,000×g for 1 h at 4°C. The supernatant was desalted using a BeyoDesalt G-25 column (P2617, Beyotime, China) and mixed with an equal volume of glycerol. The crude HPU solution was stored at 4°C for immediate use. The specific activity of the prepared HPU was 17.0 U/mg, where one unit (U) is defined as the amount of enzyme that releases 1 μmol of ammonia per minute at 37°C.

Urease inhibition experiment. A rapid urease test (RUT) was conducted to screen numerous compounds. Each compound (64 μg/mL) was added to a PBS solution containing 10 U of HPU and incubated at 37°C for 10 min. A chromogen solution containing urea (150 mM) and phenol red (30 μM) was then added, and the absorbance at 558 nm was monitored. Detailed analysis of compound-urease interactions was performed using the Berthelot method [27]. After HPU was incubated with the compound, urea (150 mM), reagent A (127 mM phenol, 0.168 mM sodium nitroprusside), and reagent B (125 mM NaOH, 0.168 mM NaOCl) were added sequentially, and the absorbance at 630 nm was monitored. For in intact bacteria testing against *H. pylori*, a bacterial suspension (4.0 × 10^7^ CFU/mL, 25 μL) was used instead of HPU. The inhibition rate is calculated using the following formula: Inhibition (%) = [1 − (A _sample_/A _control_)] × 100%, and residual activity (RA%) as RA% = (A _sample_/A _control_) × 100%. The half-maximal inhibitory concentration (IC_50_) was determined by fitting the dose–response curve of inhibition rate as a function of compound concentration.

The mode of urease inhibition by luteolin was determined through enzyme kinetic analysis. The reaction velocity (*v*) was measured at various concentrations of luteolin ([*I*]) and urea ([*S*]) using the Berthelot method [28–30]. The mode of inhibition by luteolin was determined by analyzing the changes in Michaelis constant (*K*_*m*_) and maximum velocity (*V*_*max*_) on a *Lineweaver-Burk* plot of 1/*v* vs 1/[*S*]. The inhibition constant (*Ki*) was calculated from the secondary plot of the apparent *K*_*m*_ (or *1/V*_*max*_) vs [*I*]. All experiments were conducted in triplicate.

The effects of inorganic reagents on urease activity, in the presence or absence of luteolin, were evaluated using boric acid (BA) and sodium fluoride (NaF) as reference inhibitors [29]. Specifically, urease was pre-incubated with or without luteolin in the presence of either 5 mM BA or NaF for 20 min at 37°C. Subsequently, the standard Berthelot detection reagents were added to initiate the reaction. The absorbance at 630 nm was monitored, and each assay condition was tested in triplicate.

The principle of the cellular thermal shift assay (CETSA) is to assess target engagement by monitoring ligand-induced changes in protein thermal stability [31]. In this study, crude HPU obtained from *H. pylori* lysates was incubated with 500 μM luteolin or an equal volume of DMSO (vehicle control) at 37°C for 1 h. The incubation mixture was then aliquoted into seven equal parts and subjected to heating at distinct temperatures ranging from 25°C to 75°C for 15 min, followed by cooling on ice for 5 min. After centrifugation at 15,000×g for 20 min at 4°C, the supernatant was collected, mixed with 5× SDS loading buffer, and subjected to western blot analysis to determine UreB levels. Pre-stained Protein Marker IV (8–200 kDa, G2083, ServiceBio, China) for molecular weight indication.

### Western blot

After treatment with various concentrations of luteolin for 24 h, equal amounts of *H. pylori* pellets were harvested and lysed using RIPA buffer supplemented with PMSF (1% v/v) and lysozyme. The protein concentration was determined by the BCA assay, and samples were normalized accordingly before being mixed with loading buffer. Proteins were separated by 12% Tris-glycine SDS-PAGE and transferred to PVDF membranes. Pre-stained Protein Marker IV (8–200 kDa, G2083, ServiceBio, China) was used for molecular weight indication. After blocking in 5% (w/v) skim milk, the membranes were incubated overnight at 4°C with a primary antibody against UreB (1:1,000 dilution in TBS/T, CSB-PA14359A0Rb, CUSABIO, China). Following three washes with TBS/T, the membranes were incubated with an HRP-conjugated goat anti-rabbit secondary antibody (1:1,000 dilution in TBS/T, A0208, Beyotime, China) for 2 h at room temperature. After additional TBS/T washes, protein bands were visualized using an ECL chemiluminescence substrate (G2020, ServiceBio, China) and imaged with a chemiluminescence imaging system. Band intensities were quantified by densitometry using ImageJ software and normalized to the control group. Statistical analysis was performed on the normalized data.

### Antibacterial activity and resistance assessment

The antimicrobial activity of luteolin against *H. pylori* strain ATCC43504 was evaluated. The minimum inhibitory concentration (MIC) was determined using the broth microdilution method in accordance with CLSI and EUCAST guidelines, and was defined as the lowest concentration that completely inhibited visible bacterial growth after 72 h of incubation. Following MIC determination, the minimum bactericidal concentration (MBC) was established. Briefly, aliquots from MIC assay wells showing no visible growth were plated onto a modified agar base supplemented with 10% FBS at concentrations ranging from 1/2× to 4× MIC. After 96 h of incubation under microaerophilic conditions, bacterial colony growth was visually assessed. The MBC was defined as the lowest drug concentration that resulted in a ≥99.9% reduction in the viable count compared to the control. All experiments were performed in triplicate.

For the growth inhibition assay [27] *H. pylori* was incubated with varying luteolin concentrations in modified broth containing 10% FBS. The cultures were maintained in a microaerophilic incubator for 60 h, and bacterial growth was monitored by measuring the optical density at 600 nm (OD_600_) at predetermined time points.

For the time-kill assay [27], bacterial suspensions adjusted to an OD_600_ of 1.0 were treated with varying concentrations of luteolin under the same culture conditions. Every 12 h, 50 μL aliquots were withdrawn, serially diluted, and spot-plated (10 μL) on agar plates. The plates were then incubated for 5 d at 37°C under microaerophilic conditions. The resulting bacterial colonies were counted, and the viable counts were calculated and expressed as log_10_(CFU/mL). All experiments were conducted in triplicate.

The pH shock test was performed to simulate stomach acidity and assess the inhibitory effects of compounds on *H. pylori* as described previously [29]. *H. pylori* colonies were collected, resuspended in PBS, and adjusted to a McFarland standard of 4.0. They were then mixed with different concentrations of luteolin in either 100 mM HCl (pH 1.0), with or without 5 mM urea. After 30 min in a microaerophilic environment, the samples were centrifuged (4,000×g for 10 min at 4°C), resuspended in PBS, serially diluted (10^−1^ to 10^−6^), and spot-plated on agar. After 5 d of incubation, results were calculated as log_10_(CFU/mL).

Additionally, bacterial viability under different treatments was assessed using DMAO/PI double-staining kit (C2030, Beyotime, China) according to the manufacturer’s instructions. In this assay, viable bacteria with intact membranes are stained green by DMAO, whereas bacteria with compromised membranes (necrotic) are stained red by PI. All experiments were conducted in triplicate.

The potential for resistance development to luteolin was assessed using a serial passage assay. *H. pylori* ATCC 43504 was sequentially passaged in liquid medium containing sub-inhibitory concentrations (1/2× MIC) of the compound, simulating the stepwise selection of resistant mutants. First, the MICs of luteolin and metronidazole (MTZ) against the parental strain were determined by the broth microdilution method. The strain was then inoculated into modified broth containing either 1/2× MIC or 1× MIC of luteolin or MTZ and cultured under microaerophilic conditions with shaking. Every 36 h, cultures showing growth at 1/2× MIC were subcultured into fresh medium containing the same or a higher drug concentration. Specifically, if growth occurred at the current MIC concentration, the drug concentration for the next passage was doubled. The propensity for resistance development was assessed as the fold change in MIC relative to the first passage and benchmarked against the resistance-prone antibiotic metronidazole. This assay has been adapted from a previous clinical resistance evolution model [32]. Metronidazole was selected as a clinically relevant, resistance-prone reference antibiotic, given its highest and still-rising primary resistance prevalence among first-line anti-*H. pylori* agents [3,33]. It should be noted that the reference strain ATCC 43504 is itself metronidazole-resistant [34]; the MTZ therefore served as a control to confirm that the assay could detect MIC escalation under sub-inhibitory selective pressure, rather than as a susceptibility-matched comparator. In addition, the metronidazole-resistant derivative (MR-Hp) that emerged during MTZ passaging was subsequently subjected to serial passage under sub-inhibitory luteolin in the same manner.

### Multi-omics profiling

For multi-omics profiling, paired luteolin-treated and untreated samples were prepared as described below. After 24 h of culture, *H. pylori* ATCC 43504 samples treated with or without luteolin were harvested by centrifugation. The bacterial pellets were washed twice with cold PBS (pH 7.4) and collected by centrifugation at 4,000 × g for 10 min at 4°C. The washed pellets were immediately snap-frozen in liquid nitrogen and stored at −80°C for subsequent multi-omics analysis.

Proteomic profiling was performed using a label-free, data-independent acquisition (DIA) workflow [35]. Protein extraction and quantification. Frozen *H. pylori* samples were thawed on ice and lysed in 4% SDS buffer with 1% PMSF. The lysates were heated at 95°C for 10 min, followed by sonication in an ice-water bath for 3 min. After centrifugation (12,000×g, 4°C, 5 min), the supernatant was collected. Protein quantification was determined using a BCA assay and sample quality was assessed by SDS-PAGE.

Proteolytic digestion and desalting. Equal amounts of protein from each sample were acetone-precipitated overnight at −20°C. The precipitates were washed three times with cold acetone, then resuspended and subjected to reduction with 5 mM DTT (37°C, 1 h) and alkylation with 11 mM IAA (room temperature, 45 min, in the dark). Trypsin digestion was performed overnight. The resulting peptides were desalted using C18 tips activated with 100% ACN, equilibrated with 0.1% formic acid (FA), washed twice with 0.1% FA, and eluted with 70% ACN/0.1% FA. The eluates were dried in a vacuum concentrator.

LC-MS/MS analysis. Peptide separation was performed on a Vanquish Neo UHPLC system (Thermo Scientific, USA) equipped with an EASY-Spray column (2 μm C18, 150 μm × 15 cm) maintained at 55°C. Mobile phase A was 0.1% FA in water, and mobile phase B was 0.1% FA in 80% ACN. DIA was performed on-line on an Astral mass spectrometer (Thermo Scientific, USA) with the following parameters: full MS scan range 380–980 m/z at 240,000 resolution (at 200 m/z), normalized AGC target 500%, maximum IT 5 ms, MS2 used 300 windows with 2 m/z isolation, HCD collision energy 25 eV, normalized AGC target 500%, and maximum IT 3 ms.

Data processing. Database searching was performed against the *H. pylori* UniProt proteome database (downloaded 23 January 2025). Raw data were processed using DIA-NN (v1.9.2) with the following settings, enzyme, trypsin/P, missed cleavages allowed, up to 1. Carbamidomethylation (C) was set as a fixed modification, and oxidation (M) along with protein N-terminal acetylation were set as variable modifications. Identifications were filtered at 99% protein-level confidence and a false discovery rate (FDR) ≤0.01.

Metabolomic profiling was performed using an untargeted LC-MS workflow [35]. Metabolite extraction. Bacterial samples (50 mg) were extracted with 1 mL of pre-cooled 50% methanol, flash-frozen in liquid nitrogen, thawed, and homogenized by grinding (55 Hz, 60 s). After centrifugation (12,000×g, 4°C, 15 min), 800 µL of supernatant was collected, dried under vacuum, and reconstituted in 150 µL of 50% methanol containing 5 ppm 2-chlorophenylalanine (internal standard). The solution was centrifuged again, filtered through a 0.22 μm membrane, and subjected to LC-MS analysis.

LC-MS analysis. Chromatographic separation was performed on a UPLC HSS T3 column (2.1 × 100 mm, 1.8 μm) maintained at 40°C with a flow rate of 0.4 mL/min. The mobile phase consisted of (A) 0.1% formic acid in water and (B) acetonitrile with 0.1% formic acid in acetonitrile. Data were acquired in both positive and negative ionization modes on a Thermo Scientific Q Exactive HF-X mass spectrometer operated in data‑dependent acquisition (DDA) mode. The settings were: resolution, MS1 60,000, MS2 15,000, scan range, m/z100–1,000.

Data processing. Raw data files were processed with Compound Discoverer 3.3 for peak detection, alignment, QC-based filtering, and normalization. Metabolite identification was performed by searching the mzCloud, HMDB, LIPID MAPS, MoNA, and NIST databases with mass tolerances of 15 ppm (MS1) and 50 ppm (MS2).

Bioinformatics analysis. Multivariate statistical analyses, including principal component analysis (PCA), partial least squares-discriminant analysis (PLS-DA), and orthogonal PLS-DA (OPLS-DA), were performed using the R package ropls to visualize group separations in both proteomics and metabolomic profiles. Differentially expressed proteins (DEPs) were identified from the DIA‑based quantitative proteomics data with thresholds of |fold change| >1.5 and *p*-value < 0.05. Differential metabolites (DMs) were selected based on *p*-value < 0.05 (Student’s *t*-test), |fold change| >1.2 and a variable importance in projection (VIP) score > 1 from the OPLS-DA model. Heatmaps were generated using the Pheatmap package (v1.0.12). Overlaps among comparison groups were visualized using the VennDiagram package (v1.7.3). Correlation analysis of significant differential metabolites was performed with the corrplot package (v4.0.3). Gene Ontology (GO) enrichment analysis was conducted via the Goplot (v1.0.2) using GO annotations obtained from the UniProt-GOA database. KEGG pathway enrichment analysis was implemented using the clusterProfiler package (v4.6.0), retaining pathways with *p* < 0.05. Pathway-level alterations were further evaluated by differential abundance score analysis. For integrative analysis, the KEGG pathway database was used to identify metabolic processes involving both DEPs and DMs. The metabolic network was visualized using iPath 3.0.

### In vitro cell-based Assays

The anti-invasion activity of luteolin was assessed in *H. pylori*-infected GES-1 cells. GES-1 cells were seeded in 12-well plates at a density of 5 × 10^5^ cells per well. Upon reaching confluence, the cells were washed with PBS to remove antibiotics and maintained in 1 mL of DMEM. *H. pylori* suspensions were prepared by washing with PBS and adjusting to a McFarland standard of 1.0. The GES-1 monolayers were then infected with *H. pylori* at a multiplicity of infection (MOI) of 100 and co‑cultured for 24 h under microaerophilic conditions. After washing with PBS, the cells were stained with a Calcein AM/PI double‑staining solution (C2015, Beyotime, China) at 37°C for 30 min in the dark. Cells were observed and imaged under a fluorescence microscope. In this assay, viable cells emit green fluorescence (Calcein AM), whereas dead cells emit red fluorescence (PI).

For the cell viability assay, GES-1 cells were seeded in a 96-well plate at 5 × 10^3^ cells per well. After washing with PBS, the cells were incubated in 100 µL of DMEM, infected with *H. pylori* under the same conditions for 24 h, and then assessed using a Cell Counting Kit‑8 (CCK-8, R32254, Yuanye, China). Briefly, 10 µL of CCK‑8 solution was added to each well, followed by incubation for 2 h. The absorbance at 450 nm was measured to determine cell viability.

The anti-adhesion activity of luteolin was evaluated in an *H. pylori*-GES-1 cell co-culture model. GES-1 cells were seeded in 12-well plates at a density of 5 × 10^4^ cells per well. After cell attachment, the cells were washed with PBS and refreshed with antibiotic‑free DMEM. *H. pylori* were prepared, washed, and adjusted to a McFarland standard of 1.0. The bacterial suspension was then added to the GES-1 cells at a MOI of 100 and co‑cultured for 2 h in a microaerophilic incubator.

To quantify adherent bacteria, the co‑culture was gently washed with PBS to remove non‑adherent bacteria. The remaining cells were fixed with 4% paraformaldehyde for 30 min and then stained by the Gram‑staining method for microscopic observation. In parallel, to assess bacterial viability post‑adhesion, another set of washed co‑cultures was collected, resuspended in modified broth supplemented with 10% FBS, and incubated for 24 h under microaerophilic conditions to measure the OD_600_.

For fluorescence‑based adhesion assessment, *H. pylori* were labeled with 1% FITC for 1 h at room temperature in the dark, washed, and resuspended. This FITC‑labeled bacterial suspension was used for co‑incubation with GES-1 cells for 2 h as described above. Following co‑incubation, the cells were washed, and the nuclei was counterstained with DAPI, prior to fluorescence microscopy. All experiments were performed with three independent replicates.

### In vivo studies in H. pylori-infected mice

The *in vivo* anti-*H. pylori* efficacy of luteolin was evaluated in a mouse infection model. All animal procedures were performed in accordance with protocols approved by ARRIVE guidelines and the Animal Ethics Committee of Changchun University of Chinese Medicine (Approval No. 2025803). Specific pathogen-free (SPF) male C57BL/6J mice (6 weeks old, *n* = 32) were obtained from Liaoning Changsheng Biotechnology Co., Ltd. Mice were housed under controlled conditions (temperature 25 ± 2°C, relative humidity 50 ± 5%, and a 12-h light/dark cycle). Sterile water and irradiated food were available ad libitum.

Referring to the previously reported method [36–40], in brief, after a 7-d acclimation period, the mice were randomly divided into a normal control group (*n* = 8) and a model group (*n* = 24). Mice in the model group were orally inoculated with *H. pylori* ATCC 43504 (1 × 10^8^ CFU in 0.2 mL per mouse) every 2 d for 14 consecutive days. Before inoculation, the mice were fasted and deprived of water for 6 h. Prior to infection, all mice were administered 100 μL of NaHCO_3_ (100 mM) via oral gavage, and food and water were restored 4 h post-inoculation. The control group received sterile water instead. Infection success was verified by RUT one-week post-final inoculation. Successful infection was confirmed, and infected mice were then randomly redistributed into three experimental groups (*n* = 8 per group) as outlined in Figure 7(A).

The quadruple therapy (QT) group received a standard 14-d regimen consisting of: omeprazole (5.2 mg/kg/d, fasting gavage), amoxicillin (260 mg/kg/d), potassium bismuth citrate (57.2 mg/kg/d), and metronidazole (208 mg/kg/d, gavage 1 h after feeding). The luteolin treatment group received luteolin (100 mg/kg/d) by gavage. Both the normal and model control groups received an equivalent volume of water.

Fecal *H. pylori* antigen was detected by RUT at 1‑week post‑infection (day 21) and after the final treatment (day 35) to assess eradication. After an overnight fast, blood was collected from each mouse via the eyeball extraction for serum cytokine analysis, and the animals were subsequently euthanized by cervical dislocation. The stomach of each mouse was aseptically excised, and the forestomach was removed; the remaining glandular portion was used for downstream analyses. A small piece of tissue was taken for bacterial quantification by *23S rRNA* qPCR and colony counting, using the following primers: forward, 5”-CCTTAGATTTACGGCGGATACA-3;‘ reverse, 5’-CAGTGGTAGAAGAGCGTTCATA-3.” The remaining stomach tissue, together with the adjacent duodenum, was used for histopathological examination and microbial diversity assessment.

For histopathology, stomach and duodenal tissues were processed as cross sections to allow qualitative visualization of mucosal architecture and immune cell infiltration. Tissues were fixed in 4% paraformaldehyde at room temperature for 24 h, dehydrated through a graded ethanol series, cleared in xylene, and embedded in paraffin. Sections of 1–2 μm thickness were cut using a rotary microtome, mounted on glass slides, dewaxed, rehydrated, and stained with hematoxylin and eosin (H&E) according to standard protocols. Stained sections were examined under a light microscope, and representative images were acquired from each group for qualitative comparison of mucosal morphology and inflammatory cell infiltration. Given the cross-sectional orientation, histological findings are presented as qualitative observations rather than formal pathological grading.

The gastric microbiota was characterized by full-length *16S rRNA* gene sequencing. Sample processing and sequencing were performed by Nanjing Personalbio Co., Ltd. Genomic DNA extracted from gastric tissue was amplified, and the resulting amplicons were pooled in equimolar amounts for sequencing. Single-molecule real‑time (SMRT) sequencing was conducted on the PacBio Sequel platform. To enhance accuracy, circular consensus sequencing (CCS) reads were generated from multiple subread passes. Raw CCS reads were processed using SMRT Link portal (v5.0.1.9585) with the following filters: minimum number of full passes = 3, minimum predicted accuracy = 99%. Reads exceeding 2,000 bp in length were trimmed.

Bioinformatic analysis was performed using QIIME2 (v2024.5). After demultiplexing and primer removal with the demux and cutadapt plugins, sequences were quality-filtered, denoised, and chimera-checked with DADA2 plugin. Amplicon sequence variants (ASVs) were aligned with MAFFT, and a phylogenetic tree was constructed with FastTree2. Alpha- and beta-diversity metrics were calculated to assess microbial diversity within and between samples, respectively. Beta‑diversity was visualized via principal coordinate analysis (PCoA) and hierarchical clustering (UPGMA). Taxonomic assignment of ASVs was performed with the feature‑classifier plugin using the classify-sklearn naive Bayes classifier against the SILVA *16S rRNA* gene reference database.

### Statistical analysis

All data are presented as mean ± standard deviation (SD) from at least three independent experiments. Statistical analyses were performed using GraphPad Prism (Version 10.4, GraphPad Software, LLC). For comparisons between two groups, Student’s *t*‑test (for normally distributed data) or the Mann-Whitney *U* test (for non‑normally distributed data) is applied. For comparisons among three or more groups, one‑way analysis of variance (ANOVA) followed by Dunnett’s test is used for normally distributed data, and the Kruskal–Wallis *H* test is employed for non‑normally distributed data. A *p*-value of less than 0.05 was considered statistically significant.

## Results

### Luteolin shows potential as HPU inhibitor

A two-stage screening strategy integrating molecular docking and RUT was employed to identify urease inhibitors (Figure 1(A)). A total of over 200 natural compounds were screened, with 111 successfully validated (Supplementary Data 2). Compounds with a docking score below −7 kcal/mol progressed to secondary screening, yielding a primary hit rate of 34.23%. Of these, compounds showing ≥ 60% inhibition of HPU activity within 20 min were classified as secondary hits (7.21% hit rate). The overall hit rate was 2.70%, culminating in the identification of luteolin as a promising HPU inhibitor candidate (Figure 1(B)).

### Luteolin inhibits HPU activity

The Berthelot method enables accurate quantification of urease activity by converting NH_3_, produced via urea hydrolysis, into a measurable indophenol blue complex (Figure 1(C)) [27]. Luteolin exhibited dose-dependent inhibition of HPU activity, with an IC_50_ value of 30.50 μg/mL (Figure 1(D)), consequently reducing ammonia generation detected by Berthelot method (Figure 1(E)). The HPU inhibitory effect of luteolin was further assessed in *H. pylori* (1 × 10^6^ CFU) (Figure 1(F)), showing that it suppressed ammonia production with a comparable IC_50_ of 37.54 μg/mL (Figure 1(G)). This inhibition was accompanied by a slight downregulation of UreB, as shown by western blotting, suggesting that luteolin may also interfere with urease production (Figure 1(H)).

### Luteolin competitively binds Ni^2+^-Centered catalytic site in the active pocket of HPU

**Figure 2.**
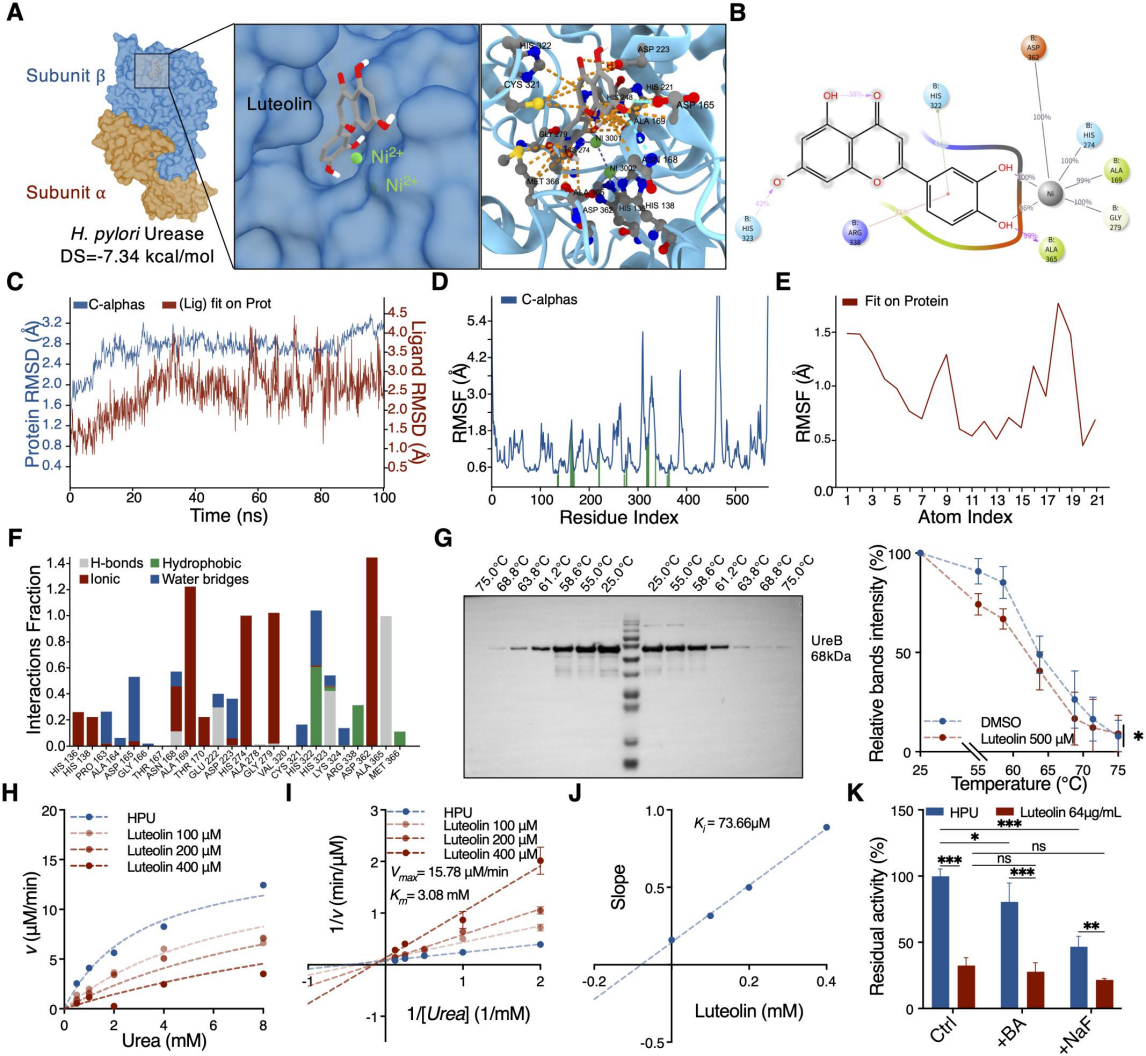
Luteolin competitively binds near the Ni^2+^ site in the active pocket of HPU. A. Molecular docking result of luteolin with HPU, showing a docking score of −7.34 kcal/mol, orange dashed lines represent hydrogen bonds. B. Interaction profile between luteolin and HPU amino acid residues, including Ni^2+^. C. RMSD of the ligand and receptor over 100 ns MD simulation, indicating stable binding conformation. D. Protein RMSF. The blue line represents the flexibility of Cα atoms for each residue, with higher values indicating greater conformational fluctuation. Green vertical bars highlight the positions of selected residues of interest. E. Ligand RMSF. The red line shows the fluctuations of ligand atoms after structural alignment to the protein binding pocket, reflecting the local mobility of the ligand within the binding site. F. Normalized summary of interactions between luteolin and HPU residues. G. CETSA validation showing altered thermal stability of HPU upon luteolin binding. Band intensity ratios relative to 25°C were used for statistical analysis (paired t-test, two-tailed, *p < 0.05). H-I. Enzyme kinetics curves and *Lineweaver-Burk* plots illustrating HPU inhibition by luteolin at varying concentrations relative to substrate inverse concentration. *V_max_* and *K_m_* are derived from the y-intercept and the negative of the x-intercept, respectively. J. Inhibition constant (*K_i_*) obtained from the slope of *Lineweaver-Burk* plots vs luteolin concentration. K. The role of Ni^2+^ in luteolin-mediated urease inhibition was evaluated using chelators (NaF and BA) via two-way ANOVA. Significance: *p* < 0.001 for Ni^2+^ chelation and HPU inhibition, *p* < 0.01 for interaction. Tukey’s multiple comparisons test: *p < 0.05, ***p < 0.001 vs HPU Ctrl, ns, *p* > 0.05 vs HPU + luteolin Ctrl, ***p < 0.001 vs HPU BA+, **p < 0.01 vs HPU NaF+. Data are from three independent experiments expressed as mean ± SD.

Given the active site of urease is situated in proximity to the Ni^2+^ ion within the HPU β subunit, molecular docking was confined to a 30 Å cubic box centered on the Ni^2+^ ion. As illustrated in Figure 2(A), luteolin precisely accommodates the pocket within the HPU β subunit, achieving a docking score of −7.34 kcal/mol. The ligand formed multiple interactions with the receptor. The Ni^2+^ ion participated in extensive interactions with luteolin, emphasizing its crucial role in stabilizing the complex (Figure 2(B)). Throughout the 100 ns molecular dynamics simulation, the root mean square deviation (RMSD) of the receptor remained stable, eventually converging between 2.8 and 3.2 Å. Similarly, after some initial fluctuation, the ligand RMSD stabilized around 3.0 Å, suggesting a consistent binding pose (Figure 2(C)). The root mean square fluctuation (RMSF) values for both the residues interacting with luteolin (Figure 2(D)) and the ligand atoms themselves fell within acceptable limits (Figure 2(E)). A hydrogen bond with ALA365 exhibited 99% occupancy, highlighting its essential role. Additional hydrogen bonds and water-mediated bridges further contributed to complex stability (Figure 2(B)).

The binding of luteolin to HPU was further confirmed by CETSA, which showed a significant shift in the thermal stability of UreB (*p* < 0.05, Figure 2(G)). Enzyme kinetic analysis employing Lineweaver-Burk plots revealed a characteristic competitive inhibition pattern, that the maximum reaction velocity (*V_Max_*) remained largely unchanged, while the Michaelis constant (*K*_m_) increased and the slope became steeper, indicating that luteolin acts as a competitive inhibitor by displacing urea from the active site of urease (Figure 2(H,I)). The inhibition constant (*K_i_*) was determined to be 73.66 μM (Figure 2(J)). To further validate the involvement of Ni^2+^ in luteolin-mediated inhibition of HPU, chelating agents including NaF and BA were employed (Figure 2(K)). Two-way ANOVA revealed a significant interaction between luteolin and Ni^2+^chelators, indicating that luteolin targets the Ni^2+^-centered catalytic site, the essential catalytic domain for urea hydrolysis in urease. This finding is consistent with the molecular dynamics simulations.

### Anti-bacterial activity of luteolin on H. pylori in vitro

**Figure 3.**
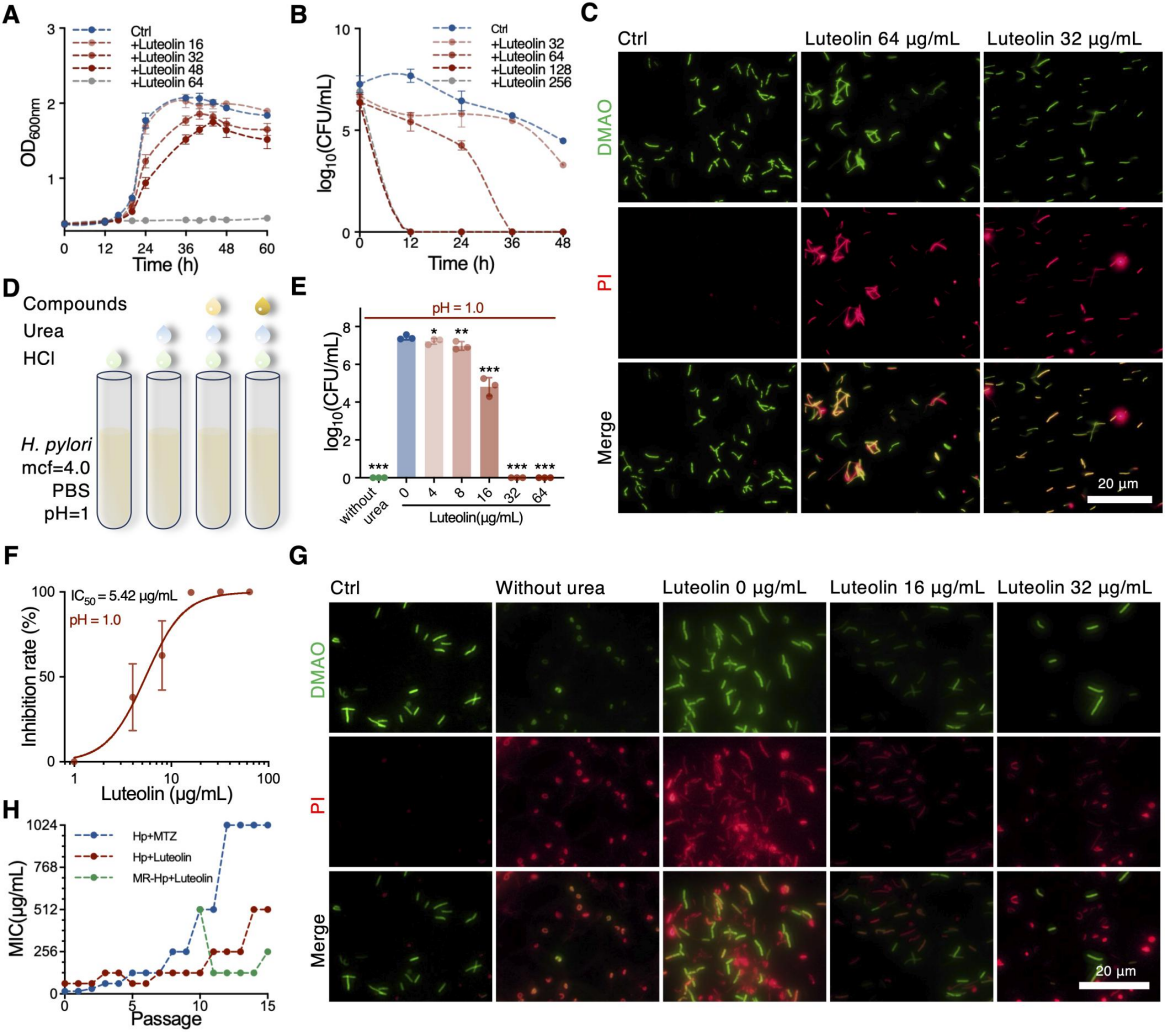
Anti-bacterial activity of luteolin against *H. pylori in vitro*. A-B. Growth inhibition kinetics and time–kill curve of luteolin against *H. pylori*. C. Fluorescence images of *H. pylori* stained with DMAO/PI after 24 h exposure to 1×MIC and 1/2×MIC of luteolin. Scale bar = 20 μm. D. Schematic diagram of the experimental setup for evaluating antibacterial activity under simulated acidic conditions. E. Bacterial colony counts expressed as log_10_(CFU/mL). *p < 0.05, **p < 0.01, ***p < 0.001 vs Luteolin 0 μg/mL. F. Anti-*H. pylori* activity of luteolin in an acidic environment, with an IC_50_ value of 5.42 μg/mL. G. Fluorescence images of H. pylori labeled with DMAO/PI staining in pH shock assay. Scale bar = 20 μm. H. Assessment of resistance development by serial passage. The metronidazole (MTZ) MIC increased 64-fold (to 1024 µg/mL) with a steep early rise (hp + MTZ), whereas the luteolin MIC increased only 8-fold (from 64 to 512 µg/mL) and only in the late passages (hp + luteolin). When the metronidazole-resistant derivative (MR-Hp) obtained during MTZ passaging was then passaged under luteolin, its luteolin MIC remained ≤ 256 µg/mL (MR-Hp + luteolin), indicating retained activity against an MTZ-resistant background. Data are from three independent experiments and presented as mean ± SD.

Luteolin exhibited an MIC of 64 μg/mL and an MBC of 128 μg/mL against *H. pylori*, as measured by standard broth dilution method [27,41]. Growth inhibition and time-kill kinetics were subsequently evaluated. It was observed that luteolin at concentrations above 32 μg/mL (1/2× MIC) significantly inhibited the growth of *H. pylori* (Figure 3(A)). In time-kill assays, luteolin at concentrations above 64 μg/mL (1/2 × MBC) exhibited strong bactericidal activity (Figure 3(B)). These quantitative results were corroborated by DMAO/PI staining, which confirmed the extensive loss of *H. pylori* viability in treated samples (Figure 3(C)).

*H. pylori* colonization of the gastric mucosa depends on urease hydrolyzing urea to produce ammonia, which neutralizes gastric acid. An *in vitro* system simulating the gastric environment was established to evaluate the anti-*H. pylori* efficacy of luteolin (Figure 3(D)). Treatment with 100 mM HCl resulted in a significant reduction of *H. pylori* colonies. In contrast, the addition of urea allowed *H. pylori* to survive, indicating that urease hydrolyzes urea to generate ammonia under urea-supplied conditions, counteracting the bactericidal effect of HCl. Under HCl challenge condition, luteolin displayed potent bactericidal effects, with 16 μg/mL significantly reducing protection and 32 μg/mL nearly completely inhibiting growth (Figure 3(E)). Moreover, the IC_50_ against *H. pylori* proliferation was determined to be 5.42 μg/mL, reflecting notable potency at a relatively low concentration (Figure 3(F)). These findings were further supported by imaging of *H. pylori* co-stained with DMAO and PI, showing a marked increase in PI-positive (dead) bacteria and a loss of viable DMAO signal (Figure 3(G)).

The emergence of resistant bacteria following unsuccessful *H. pylori* eradication therapy poses a serious clinical concern [42,43]. To evaluate the potential for resistance development, we subjected *H. pylori* strain ATCC43504 to serial passage under increasing concentrations of metronidazole (MTZ). Under sub-inhibitory selective pressure, the metronidazole MIC rose steeply and early, increasing 64-fold (from 16 to 1024 µg/mL) and plateauing by passage 12, confirming that the assay readily captures rapid resistance development in a resistance-prone agent. In marked contrast, the luteolin MIC stayed essentially unchanged through the first 10 passages and rose only gradually thereafter, reaching an ~8-fold increase (from 64 to 512 µg/mL) by passage 14, roughly eight times smaller than the escalation seen with metronidazole. Notably, when the metronidazole-resistant derivative (MR-Hp) generated during metronidazole passaging was subsequently challenged with luteolin, its luteolin MIC remained low throughout the passages (≤256 µg/mL; Figure 3(H)), demonstrating that luteolin retained activity against, and did not readily select for resistance in, an already metronidazole-resistant background. Together, these results indicate that luteolin develops resistance far more slowly and to a markedly lower degree than metronidazole, and remains effective even after metronidazole resistance has emerged.

### Targeting H. pylori urease with luteolin: a proteomic insight into pathogenicity attenuation

**Figure 4.**
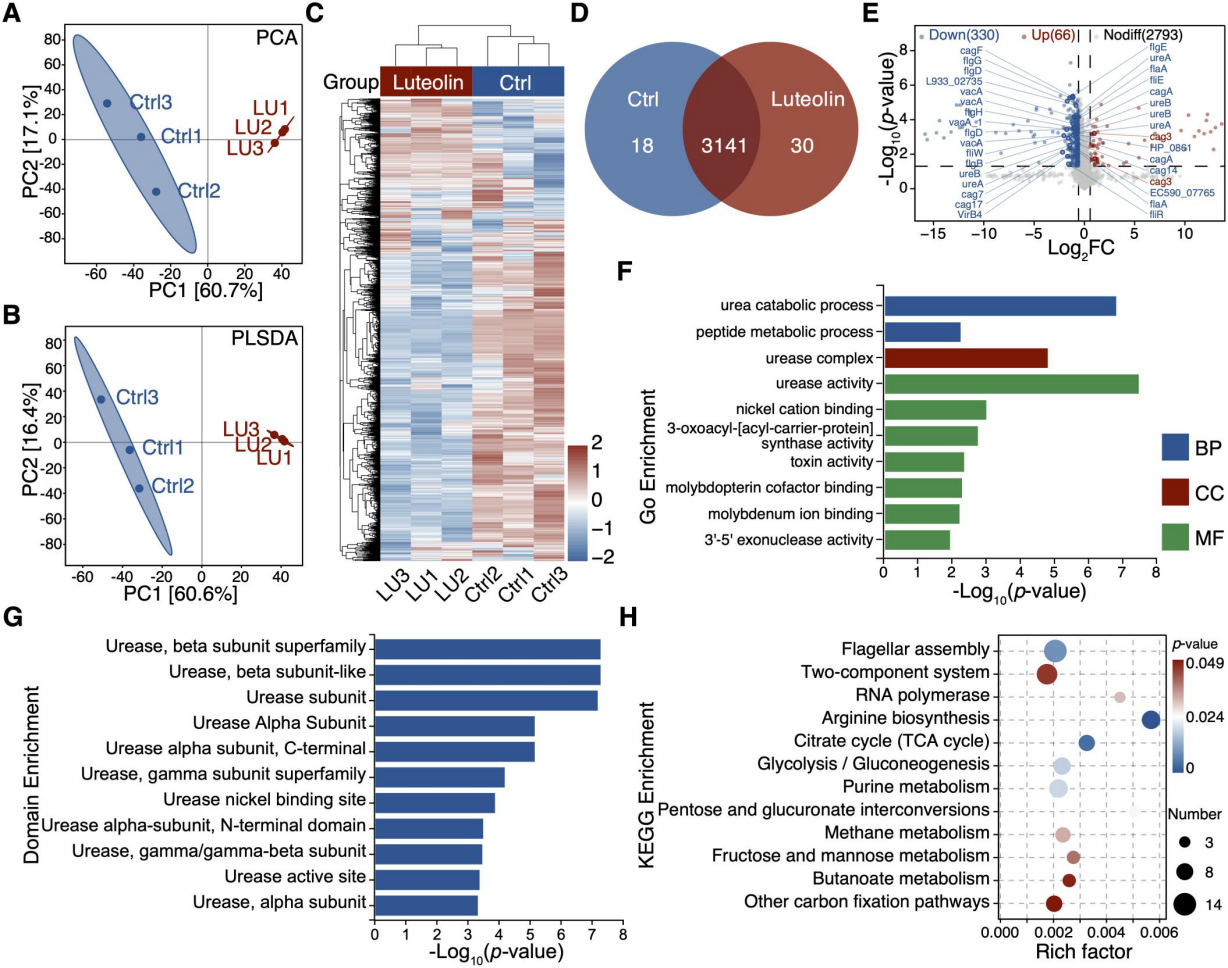
Targeting *H. pylori* urease with luteolin: a proteomic insight into pathogenicity attenuation. A-B. PCA and PLS-DA score plots demonstrate a clear separation between the luteolin-treated (LU) and control (ctrl) groups (*n* = 3). C. Hierarchical clustering analysis of all DEPs between the two groups (*n* = 3). D. Venn diagram illustrating the overlap of proteins identified in the two groups. E. Volcano plot displaying DEPs between the control and luteolin-treated groups (*n* = 3). F. Top 10 gene ontology (GO) terms enriched for the down-regulated DEPs. G. Protein domain enrichment analysis based on all DEPs. H. Top 10 KEGG pathways enriched for the down-regulated DEPs.

To elucidate the mechanisms underlying the inhibitory effect of luteolin on *H. pylori*, a proteomic analysis was performed on *H. pylori* ATCC43504 treated with luteolin (32 μg/mL, 1/2 MIC) or DMSO for 24 h. High-quality proteins and peptides were obtained (Figure 4(D)). PCA and PLS-DA revealed a distinct separation between the two groups (Figure 4(A,B)). Additionally, hierarchical clustering analysis via a global heatmap illustrated that the protein expression profile of the luteolin-treated group differed markedly from that of the control group (Figure 4(C)).

Proteomic analysis revealed 396 differentially expressed proteins (DEPs) in the luteolin-treated group compared to the control, characterized by significant changes in motility, virulence, and urease-associated defense (Figure 4(E)). Notably, key virulence factors such as CagA and VacA were downregulated. GO enrichment analysis indicated that the downregulated DEPs were significantly associated with urease-related terms, including urea catabolic process, urease activity, nickel ion binding, and the urease complex (Figure 4(F) and Supplementary Figure 1(A)). This observation was further corroborated by domain enrichment analysis (Figure 4(G)), suggesting that luteolin not only directly inhibits urease activity but may also exert a more durable inhibitory effect by disrupting the biosynthesis. Furthermore, KEGG pathway analysis revealed that the downregulated DEPs were primarily enriched in flagellar assembly, RNA polymerase, arginine biosynthesis, purine metabolism, and the TCA cycle (Figure 4(H) and Supplementary Figure S1(B)). The collective downregulation of proteins associated with motility, macromolecular biosynthesis, and metabolism implies that luteolin may induce a state of growth arrest or stress adaptation by inhibiting bacterial motility, suppressing proliferation, and impairing synthetic processes.

### Luteolin triggers ammonia restriction and global metabolic disruption in H. pylori: a metabolomic insight

**Figure 5.**
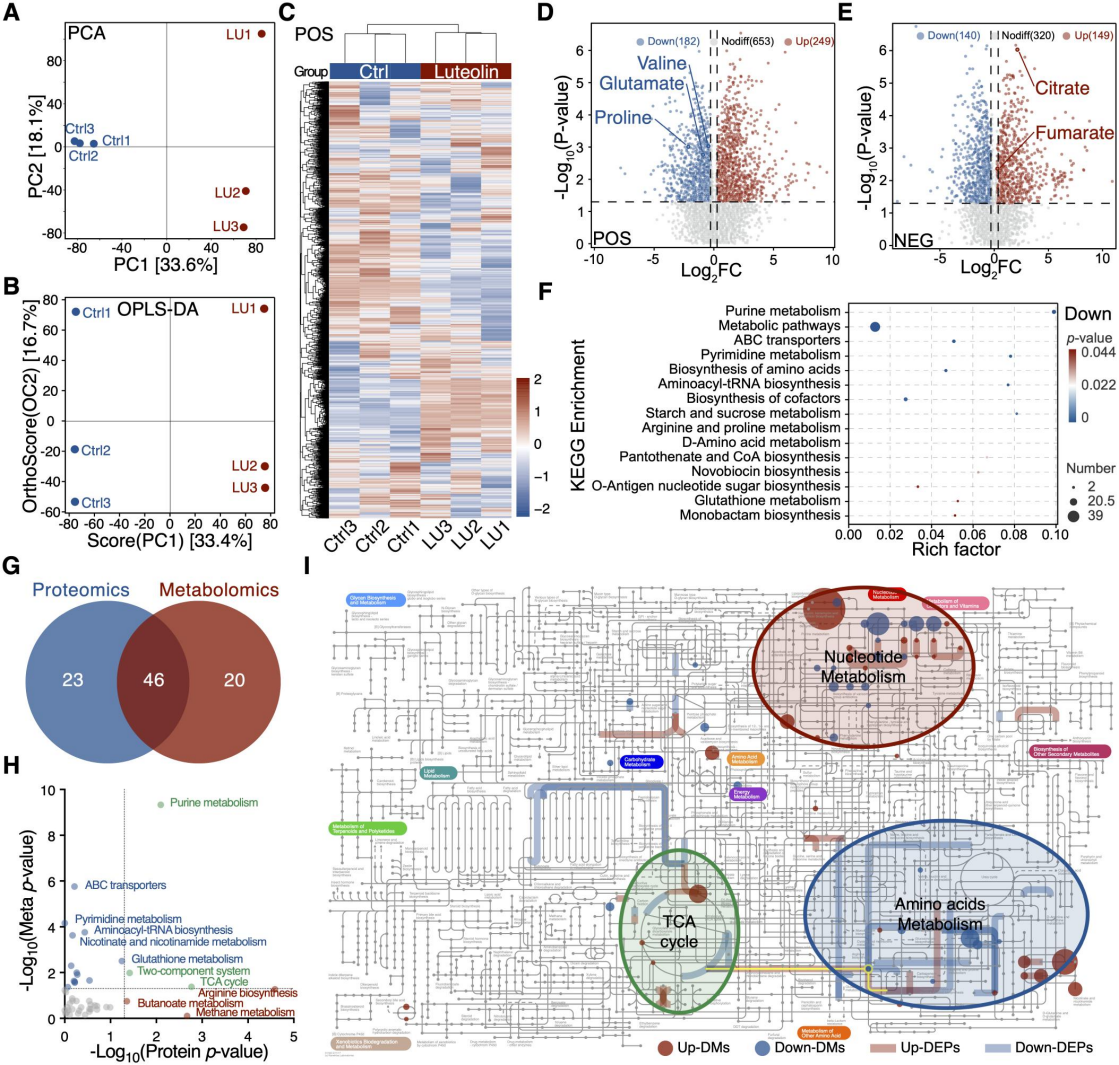
Luteolin triggers ammonia restriction and global metabolic disruption in *H. pylori*. A-B. In the positive ion mode, PCA and OPLS-DA score plots show clear separation between the luteolin-treated (LU) and control (ctrl) groups (*n* = 3). C. Hierarchical clustering analysis of DMs acquired in POS mode between groups (*n* = 3). D-E. Volcano plot of DMs between control and luteolin-treated groups. F. Top 15 enriched KEGG pathways derived from the down-regulated DEPs. G-H. Overlap and distribution of KEGG pathways identified from proteomic and metabolomic analyses. I. Integrated pathway map generated using iPath3.0, visualizing DEPs (lines) and DMs (dots). Red and blue denote up- and down-regulation, respectively. Key ammonia-assimilation enzymes are highlighted in yellow: glutamate (dot), glutamate dehydrogenase (GDH) and glutamate synthase (GOGAT, long lines), glutamine synthetase (GS) and glutaminase (GLS, short lines).

To gain deeper insights into the antimicrobial mechanism, the global metabolic profile of *H. pylori* was assessed using LC-MS-based metabolomics. Data quality and group separation were evaluated by multivariate statistical analyses, including unsupervised PCA and supervised OPLS-DA (Figure 5(A,B) and Supplementary Figure S2(A-B)). Hierarchical clustering analysis, visualized via a global heatmap (Figure 5(C) and Supplementary Figure S2(C)), revealed distinct metabolic profiles between the luteolin-treated (32 μg/mL, 1/2 MIC) and control groups. A total of 720 DMs were identified as significantly up-regulated or down-regulated (Figure 5(D,E)). Notably, glutamate levels decreased by 1.81-fold. As a central reservoir of nitrogen for many biosynthetic pathways [12], the reduction of glutamate may be attributed to the inhibition of urease activity, leading to a decreased supply of nitrogen sources for *H. pylori*.

KEGG analysis of the down‑regulated DMs revealed significant enrichment in multiple metabolic pathways dependent on urea‑derived nitrogen, specifically purine and pyrimidine metabolism, and the biosynthesis of amino acids (Figure 5(F) and Supplementary Figure S2D). Given that *H. pylori* relies primarily on urease‑generated ammonia as its nitrogen source for both acid acclimation and intracellular metabolism [12], the broad suppression of these biosynthesis pathways indicated a systemic nitrogen deficiency induced by luteolin. Therefore, by inhibiting urease activity, luteolin restricted intracellular ammonia availability, which appeared to be responsible for the subsequent disruption of nucleotide and protein synthesis, ultimately compromising bacterial viability.

Integrated multi‑omics analysis combining proteomic and metabolomic data revealed convergent pathway alterations based on KEGG enrichment analysis (Figure 5(G,H)), which were further visualized in a global metabolic map using iPath3.0 (Figure 5(I)). In summary, our findings demonstrate that luteolin potently inhibits urease activity, thereby restricting the intracellular ammonia supply in *H. pylori*. This restriction inhibits the acid‑neutralizing capacity of the bacterium and concurrently triggers a compensatory generation of ammonia from amino acids and amides. Consequently, this adaptive response disrupts amino acid metabolic homeostasis, leading to extensive dysregulation of fundamental metabolic pathways, including nucleotide and amino acid biosynthesis, ultimately resulting in bacterial death.

### Luteolin protects GES-1 cells against H. pylori-induced damage

**Figure 6.**
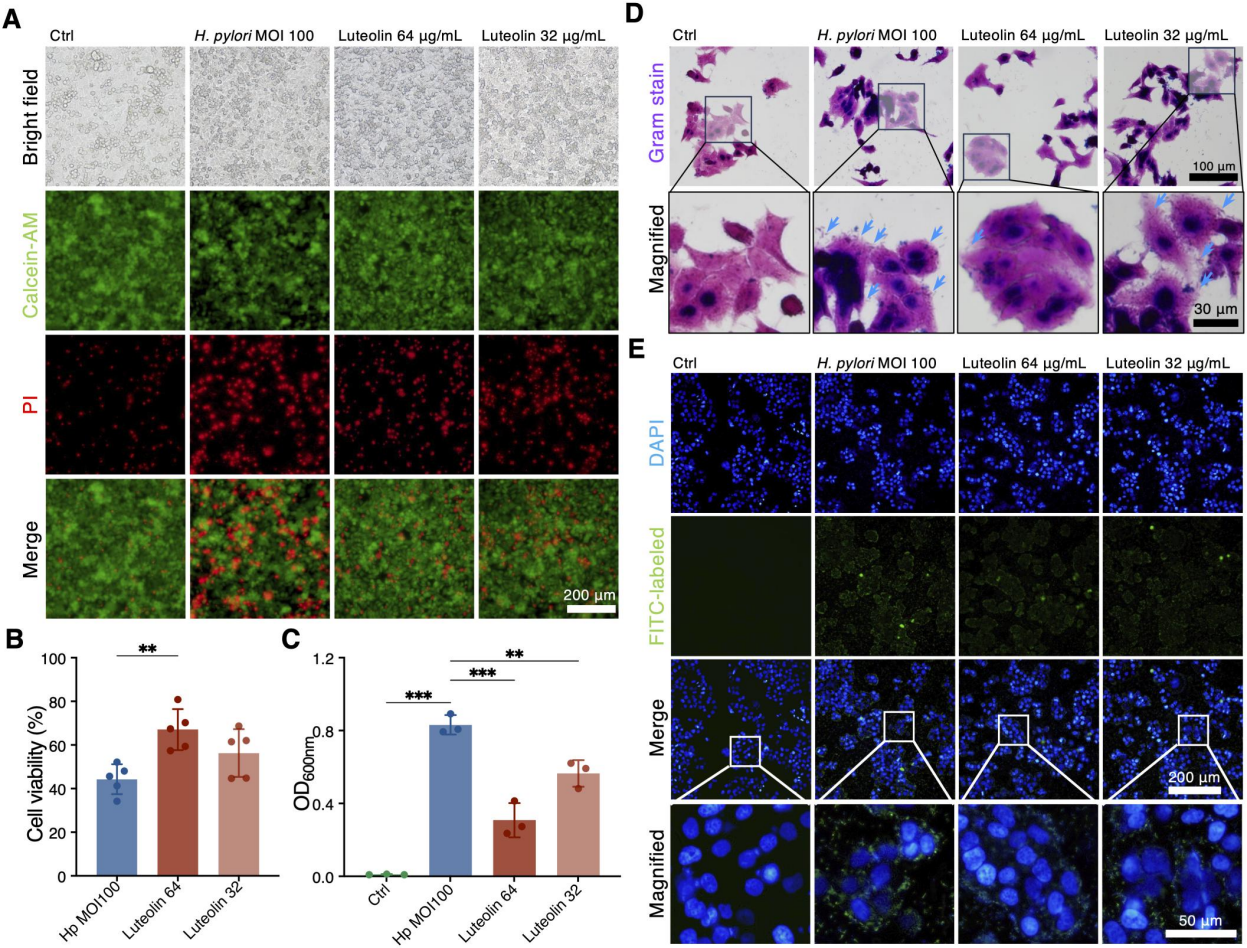
Luteolin protects GES-1 cells against *H. pylori*-induced damage. A-B. Evaluation of GES-1 cells after 24 h infection with *H. pylori*. A. Cell status labeled with Calcein-AM (live, green) and PI (dead, red). Scale bar = 200 μm. B. Cell viability assessed by CCK-8 assay. **p < 0.01 vs GES-1 + *H. pylori* (MOI 100). C-E. Assessment of *H. pylori* status after 2 h of infection. C. Quantification of adherent bacteria. **p < 0.01, ***p < 0.001 vs GES-1 + *H. pylori* (MOI 100). D. Gram staining showing adherent *H. pylori*(blue arrows). Scale bars = 100 μm (overview) and 30 μm (magnified view). E. Fluorescence imaging of FITC-pre-labeled *H. pylori* (green) and DAPI-labeled GES-1 cells (blue) in co-cultures. Scale bar = 200 μm (overview) and 50 μm (magnified view).

The cytoprotective potential of luteolin was evaluated *in vitro*. GES-1 cells infected with *H. pylori* (MOI 100, 24 h) exhibited extensive cell death observed by Calcein-AM/PI staining. This *H. pylori*-induced cytotoxicity was markedly attenuated by luteolin treatment (Figure 6(A)). Subsequently, cell viability was quantified using the CCK-8 assay. In the *H. pylori*-infected group, cell viability decreased to 44.30 ± 6.87%. In contrast, luteolin treatment significantly restored viability to 67.10 ± 9.37% (64 μg/mL) and 56.30 ± 11.00% (32 μg/mL), respectively (Figure 6(B)). These results demonstrated that luteolin exerts a protective effect on GES-1 cells against *H. pylori*-induced damage.

HPU plays a role in the process of *H. pylori* adhesion, which is a key step in colonization [44]. To visualize bacterial adhesion, GES-1 cells were infected with *H. pylori* (MOI 100). After 2 h of co-culture, the cells were fixed and Gram-stained. Compared to the control group, the infected group showed numerous stained rods adherent to the GES-1 cell surface (Figure 6(D)). Treatment with luteolin reduced the number of adhering *H. pylori*. In a separate assay, adherent bacteria were quantified (Figure 6(C)). Compared with the infection group, the relative bacterial attachment in the luteolin-treated group decreased from 0.83 ± 0.05 to 0.31 ± 0.09 or 0.57 ± 0.07 (OD_600_, 64 μg/mL, *p* < 0.001 or 32 μg/mL, *p* < 0.01). In adhesion assays with FITC-labeled *H. pylori*, luteolin treatment markedly decreased green fluorescence (adhered bacteria, Figure 6(E)), indicating luteolin reduced *H. pylori* adhesion.

### Luteolin reduces bacterial load and alleviates H. pylori-induced mucosal damage

**Figure 7.**
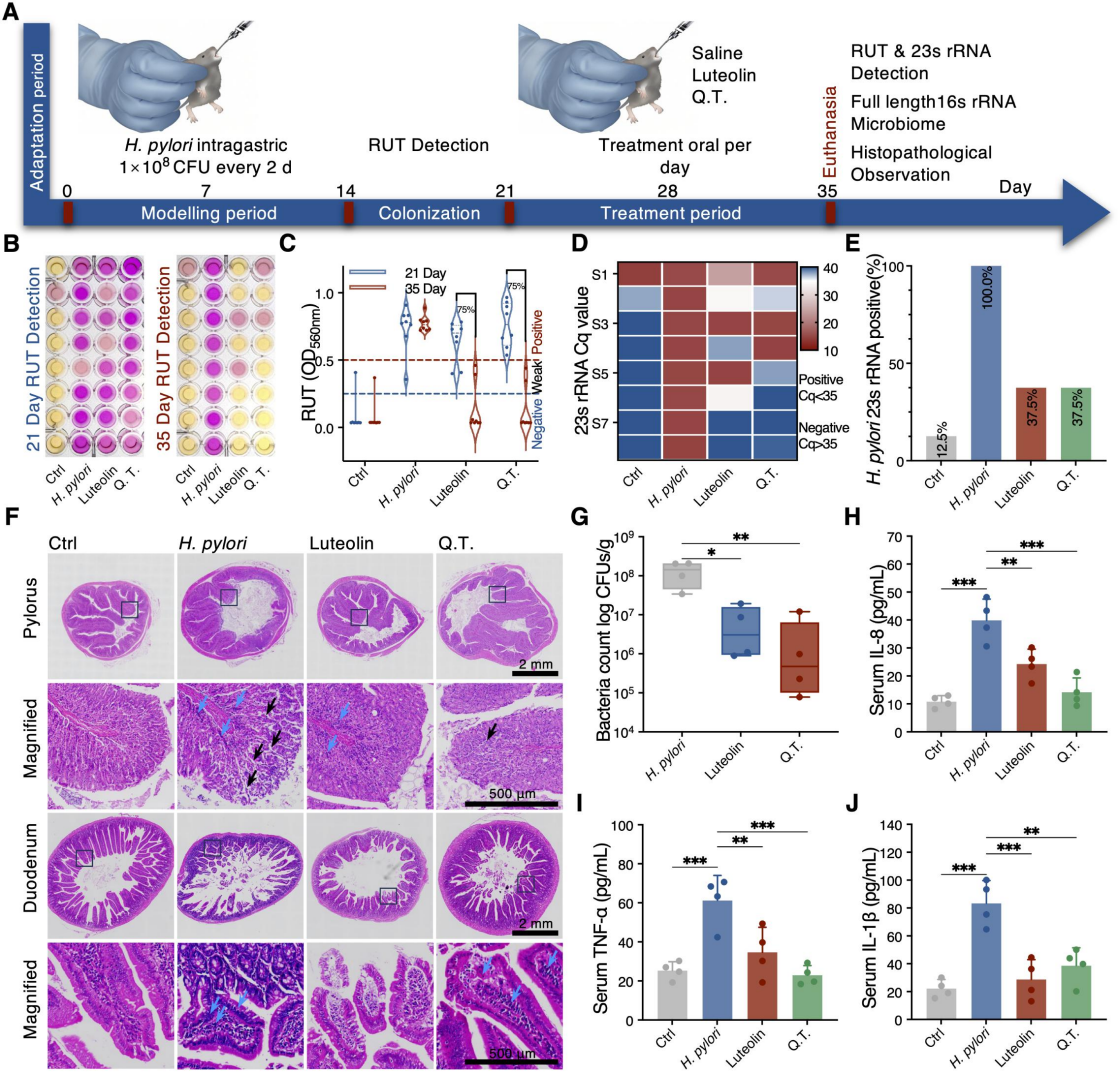
Luteolin reduces bacterial load and alleviates *H. pylori*-induced mucosal damage. A. Experimental timeline for *H. pylori* infection in C57BL/6J mice. QT, quadruple therapy. B-C. RUT of mouse fecal samples post-colonization and post-treatment, quantified by OD_560_ (*n* = 8). Results were classified as positive (>0.5), negative (<0.25), or weakly positive (0.25–0.5). D-E. *H. pylori* eradication assessed by RT-qPCR detection of *23S rRNA* in gastric tissues (*n* = 8). A sample was considered positive (cq < 35) or negative (cq > 35). F. Representative H&E-stained sections of the gastric pylorus and duodenum, serve as a qualitative reference to visualize immune cell infiltration and overall mucosal architecture. Black arrows indicate widened intercellular spaces and less organized cell arrangement. Blue arrows indicate inflammatory cells. Scale bars = 2 mm (overview) and 500 μm (magnified view). G. Bacterial load in mouse gastric homogenates determined by colony counting on selective medium (*n* = 4). H-J. Serum levels of pro-inflammatory cytokines (*n* = 4). *p < 0.05, **p < 0.01,***p < 0.001 vs *H. pylori* group.

A *H. pylori* infection mouse model was established by oral inoculation with *H. pylori* (1 × 10^8^ CFU per dose) every other day over a 14‑d period [27] (Figure 7(A)). Fresh fecal samples collected on day 21 for RUT showed an infection rate of approximately 87.50%, while weak positivity was detected in the remaining 12.50% (Figure 7(B)). After 14 consecutive days of treatment, the eradication rate of *H. pylori* in the luteolin-treated mice reached 75.00% (Figure 7(C)). The RUT findings were validated using a qPCR assay specific for the *H. pylori 23S rRNA* gene on gastric tissue samples (Figure 7(D)). Utilizing a Cq value of less than 35 as the threshold for *H. pylori* positivity, luteolin group showed a positivity rate of 37.50% among the eight samples, corresponding to an eradication rate of 62.50% at the tissue level (Figure 7(E)).

Histopathological examination of the mouse digestive tract was performed on H&E-stained sections of pyloric and duodenal tissues (Figure 7(F)), which were used to provide a qualitative visualization of mucosal morphology and immune cell infiltration rather than formal pathological grading. Compared to the generally intact mucosal architecture observed in control animals, representative sections from the *H. pylori*-infected model group appeared to show widened intercellular spaces, less organized cell arrangement (black arrow), apparent inflammatory cell infiltration extending into the submucosa (blue arrow). Neutrophil infiltration (blue arrow) was also visible in the duodenal mucosa. Both luteolin and standard quadruple therapy qualitatively reduced these histological changes, with a noticeable decrease in inflammatory cell infiltration (blue arrow). In the luteolin-treated group, glandular architecture appeared closer to that of the control animals, while sections from the quadruple therapy group showed a comparatively looser glandular organization (black arrow). Together with the bacterial colonization and inflammatory cytokine data presented above, these observations are consistent with luteolin mitigating *H. pylori*-induced mucosal damage within the digestive tract.

The bacterial load in gastric tissue homogenates was quantified by colony counting on selective agar (Figure 7(G)). In the *H. pylori*-infected group, the load reached 1.38 × 10^8^ CFU/g. Treatment with luteolin significantly reduced this load to 7.52 × 10^6^ CFU/g (*p* < 0.05), slightly less potent than that of quadruple therapy (3.33 × 10^6^ CFU/g, *p* < 0.01). Furthermore, serum levels of key cytokines were assessed (Figure 7(H-J)). *H. pylori* infection elevated levels of IL‑8 (KC), IL‑1β, and TNF‑α. Both luteolin and quadruple therapy significantly suppressed these cytokines (*p* < 0.01 or *p* < 0.001), demonstrating comparable anti‑inflammatory efficacy.

### Microbiota analysis: luteolin-maintained gastric microbiota homeostasis

**Figure 8.**
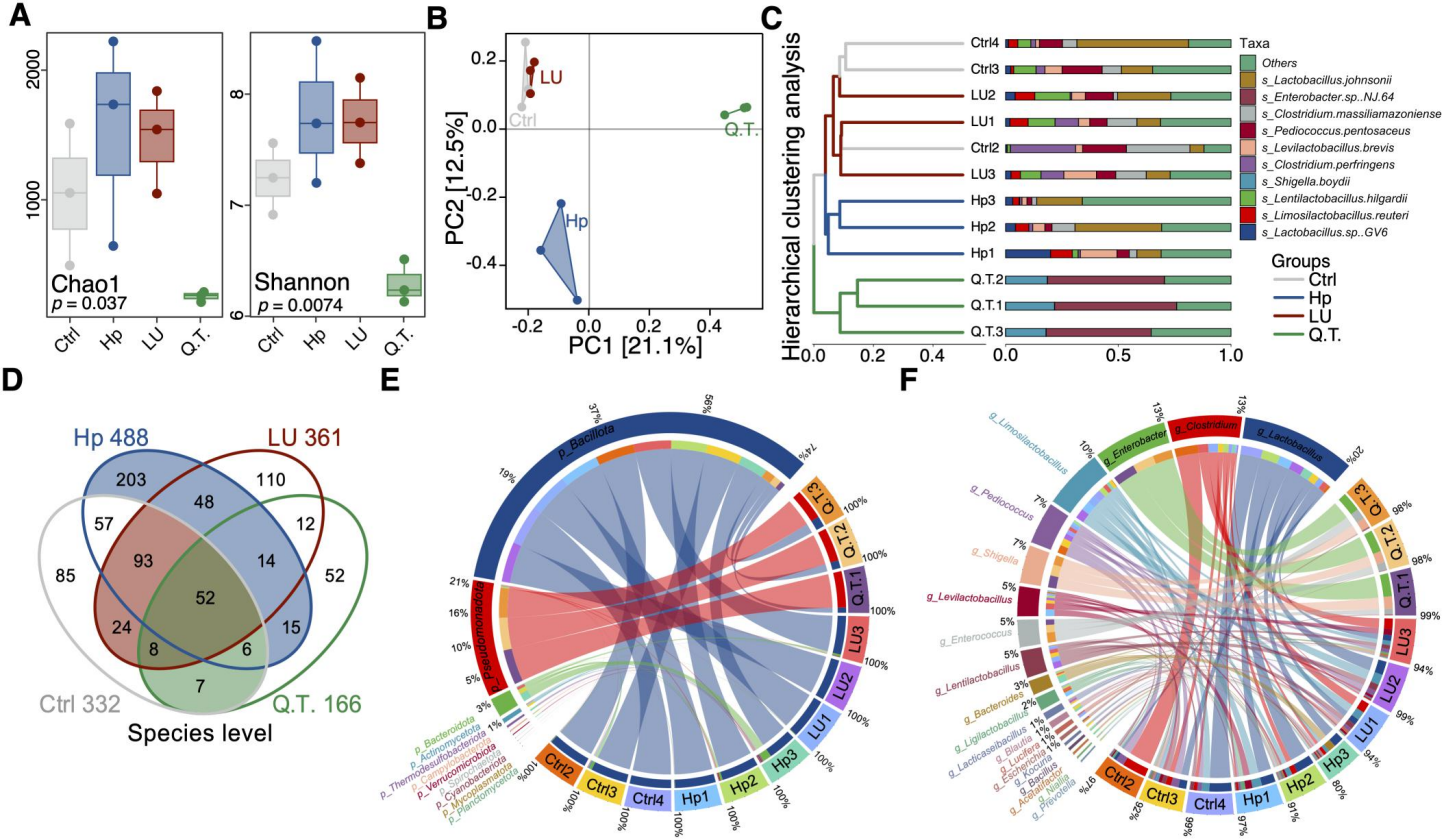
Luteolin-maintained gastric microbiota homeostasis. A. Alpha-diversity analysis. Microbial diversity within groups was assessed using the Chao1 index (richness) and Shannon index (diversity). Antibiotic treatment significantly reduced both richness and evenness. Ctrl, control group. Hp, *H. pylori*-infected group. LU, luteolin-treated group. QT, quadruple therapy group. B-C. Beta-diversity analysis. Principal coordinates analysis (PCoA) based on Bray-Curtis distance (B) and hierarchical clustering (C) reveal distinct segregation among groups, indicating that antibiotic treatment significantly alters the overall composition of the gastric microbiota. D. Venn diagram illustrating the distribution of species across groups. E-F. Species composition analysis and chord diagrams at different taxonomic levels (E, phylum level, F, genus level).

Full-length *16S rRNA* sequencing was performed to profile the gastric microbiota. Alpha-diversity analysis revealed that the Chao1 (richness) and Shannon (evenness) indices were significantly lower in the quadruple therapy (QT) group than in controls (ANOVA, *p* < 0.05, Figure 8(A)), indicating a marked reduction in microbial diversity. Beta-diversity analysis based on Bray-Curtis distances showed a clear separation in community structure, with samples from the QT group clustered apart from the control and luteolin groups, while the latter two largely overlapped in principal coordinate analysis (PCoA, Figure 8(B)). Consistent with this, the QT group exhibited greater dispersion in community structure, reflecting severe dysbiosis and high inter‑individual variability (Figure 8(C)).

The Venn diagram illustrated distinct species overlaps with the control group (Figure 8(D)). The *H. pylori*-infected group contained the most unique species (blue area, *n* = 280) and shared only 208 species (62.65%) with controls. Quadruple therapy severely reduced species overlap, retaining only 73 species (green area, 21.99% of control) and yielding the lowest total species count. In contrast, luteolin treatment showed a substantially higher overlap (177 shared species, red area, 53.31% of control), indicating a much milder impact on the resident microbiota.

At the phylum level, Bacillota, Pseudomonadota, Bacteroidota, and Actinomycetota were predominant (Figure 8(E)). Bacillota accounted for over 90.00% of sequences in all groups except the antibiotic group, where its abundance declined sharply and was replaced by Pseudomonadota (80.60 ± 6.34%). Bacteroidota and Actinomycetota were nearly eradicated after antibiotic treatment.

Genus-level analysis revealed that *Lactobacillus*, a probiotic genus, was predominant in controls and increased upon *H. pylori* infection, consistent with prior reports [45]. Antibiotic treatment nearly eliminated *Lactobacillus* and promoted the expansion of *Enterobacter*, *Enterococcus*, and *Shigella* (Figure 8(F)). The emergence of opportunistic pathogens like *Shigella* aligns with known antibiotic‑resistance patterns [46]and may reflect altered gastric conditions post‑therapy [47]. In contrast, the luteolin group maintained a genus profile similar to controls.

In summary, quadruple therapy effectively but non-selectively eradicated gastric microbiota, leading to severe dysbiosis. In contrast, luteolin treatment preserved microbial community structure and diversity, resembling that of healthy controls.

## Discussion

*H. pylori*‑associated gastric cancer remains a global burden, and antibiotic resistance now threatens standard therapies [3,48], with reported resistance reaching 30–61% for key antibiotics [3,4]. This pressing issue necessitates the exploration of alternative therapeutic strategies. Consequently, our work pursued a resistance-avoidant approach by targeting bacterial virulence with non‑antibiotic agents.

Despite the report of hundreds of HPU inhibitors, the development has been constrained by challenges in achieving sufficient in *vivo* potency and selectivity [49]. In the present study, integrated multi-omics analyses revealed that luteolin acts as a HPU inhibitor. By precisely targeting HPU, luteolin effectively blocks ammonia production in *H. pylori*, thereby compromising its acid-neutralizing capacity, which represents a key defensive mechanism of the bacterium. This disruption extends to the intracellular metabolic homeostasis, leading to dysregulation of amino acid and nucleotide metabolism. The convergence of extracellular defense impairment and intracellular metabolic dysfunction culminates in the irreversible collapse of bacterial viability.

Metabolomic results indicated that urease inhibition triggered a metabolic reprogramming in *H. pylori*. Under the ammonia-limited state induced by urease inhibition, glutamate is compelled to release NH_3_ via GDH to counteract the threat of protons. The upregulation of glutamine synthetase (GS) confirms the current glutamate synthesis mode under low NH_3_ levels. The unexpected upregulation of glutamate dehydrogenase (GDH), which should remain silent in the glutamate synthesis mode under low NH_3_ levels, suggests that this reversible reaction is releasing ammonia from the storage pool. Following urease inhibition, *H. pylori* was compelled to exploit glutamate and glutamine as alternative ammonia sources, triggering a metabolic compensation. This emergency response depleted cellular glutamate pool, which is essential for amino acid recycling and nucleotide biosynthesis. The resulting glutamate scarcity consequently starved multiple anabolic pathways. Simultaneously, the accumulated 2-oxoglutarate impaired TCA cycle function. Collectively, disruptions in nitrogen supply, biosynthetic capacity, and energy metabolism synergized to create an irrecoverable metabolic crisis that directly contributed to bacterial death.

*H. pylori* pathogenesis depends not only on colonization but also on virulence factors like CagA and VacA, which directly exacerbate gastric damage and inflammation [50]. Therefore, an ideal agent would both hinder bacterial survival and mitigate virulence. Here, luteolin-mediated HPU inhibition was accompanied by reduced CagA and VacA expression, suggesting a mechanism beyond direct enzymatic blockade. Although this could partly reflect reduced bacterial fitness, two observations favor a more direct link: sub-inhibitory concentrations of small molecules can coordinately downregulate *cagA*, *vacA* and *ureA/B/I* without measurably impairing *H. pylori* growth [51]; urease-derived ammonia is the major nitrogen donor for amino-acid synthesis [52], consistent with the amino-acid metabolic perturbations observed here. We interpret CagA/VacA suppression as predominantly a direct downstream consequence of urease inhibition, though a contribution from reduced bacterial fitness cannot be entirely excluded. This dual mechanism concurrently disrupts acid resistance through ammonia restriction and mitigates host cell injury by attenuating virulence, thereby collectively impairing bacterial survival and pathogenicity. Luteolin therefore emerges as a promising anti-*H. pylori* candidate with multiple mechanisms, offering integrated therapeutic benefits.

Given the increasing challenge of *H. pylori* antibiotic resistance and the recognized adverse effects of current therapies on gastric microbiota, luteolin demonstrated a favorable therapeutic profile. It achieved a bacterial clearance effect comparable to that of standard quadruple therapy while alleviating mucosal inflammation and tissue injury. Notably, luteolin preserved the gastric microbiota, maintaining its diversity and ecological balance, in contrast to the severe dysbiosis induced by conventional antibiotics. Furthermore, compared with metronidazole, luteolin showed an 8-fold smaller and markedly slower increase in MIC under serial passage, and remained effective even against a strain that had already acquired metronidazole resistance. These characteristics support the potential of luteolin as a promising candidate to overcome key limitations in existing *H. pylori* treatment strategies.

Studies have shown that luteolin enhances the resistance of *Caenorhabditis elegans* to a broad spectrum of clinically relevant pathogens [17], inhibits MRSA biofilm formation and cytotoxicity by suppressing bacterial toxin biosynthesis [18], and disrupts cell wall and membrane integrity in multidrug-resistant *Escherichia coli*, ultimately resulting in bacterial lysis and death [19]. Beyond its direct bactericidal effects, luteolin is capable of attenuating or reversing bacterial drug resistance via distinct molecular mechanisms, including inhibition of key bacterial enzymes, suppression of efflux pumps, impairment of energy metabolism, and disruption of cell wall integrity to impede biofilm formation [16]. To date, however, the majority of studies on luteolin remain at the preclinical stage, and large‑scale, rigorous randomized controlled trials have yet to be conducted [53]. In preclinical models, the median lethal dose (LD_50_) of luteolin exceeds 2500 mg/kg in mice and 5000 mg/kg in rats, and luteolin at concentrations of 25–100 μg/mL for 24 h demonstrated no cytotoxic effect against intestinal epithelial cell lines [53]. The gastroprotective value of luteolin appears to extend beyond its antimicrobial properties. Emerging evidence suggests that luteolin may also directly modulate the gastric mucosa to counteract pre‑neoplastic changes, such as oxyntic atrophy and spasmolytic polypeptide-expressing metaplasia [54]. This is further supported by independent research highlighting the translational promise of luteolin in the context of gastric cancer therapy [55]. Collectively, the available evidence indicates that luteolin may exert synergistic protective effects against *H. pylori*-associated gastric carcinogenesis, through both its antimicrobial activity and concurrent mucosal protection. A limitation of the present study is that, although oral luteolin produced robust anti*-H. pylori* efficacy in vivo, the local luteolin concentration achieved at the gastric mucosa was not directly measured; site-specific pharmacokinetic profiling and formulation optimization (such as nanocarriers, phosphate prodrugs, or gastroretentive systems [56–58]) are warranted to fully define and enhance its translational potential.

A limitation of the resistance-development assay is that the comparator antibiotic, metronidazole, was tested in a strain (*H. pylori* ATCC 43504) with preexisting metronidazole resistance; although luteolin’s own low resistance propensity and its retained activity against the resistant strain are unaffected by this, future studies employing a susceptible-baseline comparator (e.g. clarithromycin) would further substantiate the comparative resistance dynamics.

In conclusion, luteolin is a natural flavonoid that competitively inhibits *H. pylori* urease, thereby blocking urea-derived ammonia production. This impairs bacterial acid resistance and metabolism, leading to effective suppression of the infection and reduction of gastric damage. Notably, luteolin presents a low risk of inducing resistance and causes minimal disruption to gastric microbiota, highlighting its promise as a non-antibiotic therapy.

## Supplementary Material

Supplementary Figure 1 without legend.tif

Supplementary Figure 2 without legend.tif

Supplementary Data 2.xlsx

Supplementary Figure Legends.docx

## Data Availability

The data and supplementary data that support the findings of this study are openly available in figshare at https://doi.org/10.6084/m9.figshare.31398882, reference number. The mass spectrometry proteomics data have been deposited to the ProteomeXchange Consortium via the PRIDE partner repository with the dataset identifier PXD072422 (https://www.ebi.ac.uk/pride/archive/projects/PXD072422). The mass spectrometry based metabolomics data have been deposited to the Metabolights database with the dataset identifier MTBLS13581 (https://www.ebi.ac.uk/metabolights/MTBLS13581). The Microbiota Analysis sequencing data generated in this study have been deposited in the National Center for Biotechnology Information Sequence Read Archive under the BioProject accession number PRJNA1394526 (https://www.ncbi.nlm.nih.gov/sra/PRJNA1394526).
