# Supplementary material for: Luteolin as a urease inhibitor: A novel therapeutic strategy against Helicobacter pylori
Source: Virulence. 2026 Aug 3;17(1):2712031. doi: 10.1080/21505594.2026.2712031 (PMC13449824; doi:10.1080/21505594.2026.2712031)
Supplement: Supplementary file 1 [file KVIR_A_2712031_SM4887.docx]

**Supplementary Figure Legends**

**Fig.S1 Targeting H. pylori Urease with Luteolin:** A Proteomic Insight into Pathogenicity Attenuation. A. Top 10 Gene Ontology (GO) terms enriched for the up-regulated DEPs. B. Top 10 KEGG pathways enriched for the up-regulated DEPs.

**Fig.S2 Luteolin Triggers Ammonia Restriction and Global Metabolic Disruption in *H. pylori*.** A-B. In the negative ion mode, PCA and OPLS-DA score plots show clear separation between the luteolin-treated (LU) and control (Ctrl) groups (n = 3). C. Hierarchical clustering analysis of DMs acquired in NEG mode between groups (n = 3). D. Top 10 enriched KEGG pathways derived from the up-regulated DEPs.
